# 4142 Implementation of Consent-to-Contact (CTC) initiative at an Academic Medical center: Initial operationalization and lessons learned

**DOI:** 10.1017/cts.2020.238

**Published:** 2020-07-29

**Authors:** Chin Chin Lee, Helenmarie M. Blake, Carlos A. Canales, Stephen J. DeGennaro, Ishwar Ramsingh, Daru Lane Ransford, Carl I. Schulman, Jonelle Wright, Ralph L. Sacco

**Affiliations:** 1 University of Miami Clinical and Translational Science Institute; 2 University of Miami Miller School of Medicine

## Abstract

OBJECTIVES/GOALS: The objectives of this presentation are to discuss 1) the implementation of Consent to Contact at an Academic Medical Center; 2) the access to lists of potential participants by study teams; and 3) the challenges and adjustments made to the initial conceptualized process. METHODS/STUDY POPULATION: Participant recruitment is critical to the success of all research studies. It is particularly challenging when investigators do not have a patient population from which to recruit. Thus, the University of Miami launched the CTC initiative in 2016 to facilitate study recruitment. Study investigators can request access to a registry of participants who agreed to be contacted and meet the initial study eligibility criteria. A multidisciplinary Operational Committee provides oversight and regulates access to the CTC registry. RESULTS/ANTICIPATED RESULTS: The registry has over 110K patients who have agreed to be contacted for eligible research studies. The demographic distribution of the patients in the registry mirrors the diversity of the UHealth population. As of January 2018, when the registry became available to the research community, 25 study teams from different departments, including the All of Us Research Program, have requested potential participant lists. The process of requesting access to patient lists is adapted to studies’ needs, with particular reference to sensitive populations, such as HIV/AIDS, substance abuse, etc. Results on utilization and satisfaction of the CTC initiative are being collected and will be presented. DISCUSSION/SIGNIFICANCE OF IMPACT: The CTC initiative allows UHealth patients to opt-in to the registry for research studies. The Operational Committee continues to monitor the successful consent of patients to participate in individual research studies and improving the request process.

